# Boosting Capacitive Deionization Performance via Bimetallic Synergistic Engineering of Electrospun Co/N-Doped Porous Carbon Nanofibers

**DOI:** 10.3390/membranes16070243

**Published:** 2026-07-17

**Authors:** Xinyue Ma, Yuan Li, Kuo Meng, Chengbo Kou, Binling Li, Zhonglei Zhu, Haojie Li, Zhihan Deng, Runze Yang, Hupeng Zhou, Xin Wang, Lang Luo, Fuming Chen, Chengding Gu, Yuxiao Zhang, Lu Guo

**Affiliations:** 1 School of Materials and Energy, Yunnan University, Kunming 650091, China; maxinyue@stu.ynu.edu.cn (X.M.); leey@ynu.edu.cn (Y.L.); mengkuo@stu.ynu.edu.cn (K.M.); k15733776531@163.com (C.K.); libinling1@stu.ynu.edu.cn (B.L.); 12023235156@stu.ynu.edu.cn (H.L.); 12024235024@stu.ynu.edu.cn (Z.D.); yangrunze@stu.ynu.edu.cn (R.Y.); zhouhupeng@stu.ynu.edu.cn (H.Z.); wangxin20040305@163.com (X.W.); 12025235100@stu.ynu.edu.cn (L.L.); chengdinggu@ynu.edu.cn (C.G.); 2 Yunnan Malaya Institute, School of engineering, Yunnan University, Kunming 650091, China; fmchen@m.scnu.edu.cn; 3 Yunnan Key Laboratory of Electromagnetic Materials and Devices, School of Engineering, Yunnan University, Kunming 650091, China

**Keywords:** capacitive deionization, electrospinning, porous carbon fibers, cobalt/nitrogen doping

## Abstract

Capacitive deionization (CDI) is an environmentally advantageous desalination technology that is particularly suitable for the treatment of low- to medium-concentration saline water. Among different electrode materials, carbon materials are widely used due to their good electrical conductivity and high specific surface area, while they suffer from limited ion adsorption capacity. In this study, a cobalt/nitrogen-doped porous carbon fiber composite with Zn-induced porosity (CoNG@V@CNF), where “V” stands for “volatile pore-forming agent”, has been successfully prepared via electrospinning combined with a high-temperature carbonization process. The introduction of trace Co nanoparticles enhances the stability of porous graphene. In addition, N doping contributes to improved wettability and electronic conductivity, and the carbon fiber structure constructs a three-dimensional conductive network, providing fast channels for ion transport. Electrochemical tests show that the specific capacitance of CoNG@V@CNF reaches 252.76 F g^−1^, demonstrating its superior charge storage capability. Furthermore, this study achieved a high salt adsorption capacity of 58.28 mg g^−1^ and a competitive desalination rate performance of 1.94 mg g^−1^ min^−1^. After 40 cycles of testing, the salt adsorption capacity (SAC) remains at 56.72 mg g^−1^, demonstrating its high stability during multiple charging and discharging processes. This work provides a new design strategy for developing high-performance CDI electrode materials.

## 1. Introduction

Water resources are the foundation for the survival and development of human society. However, with population growth, rapid economic development, and the impacts of climate change, the global shortage of freshwater resources is becoming increasingly serious [1]. The situation regarding water resources in China is particularly severe, with per capita water resources far below the world average and an extremely uneven spatiotemporal distribution, making it difficult to meet the water demands for sustainable socio-economic development [2]. Since ocean water resources are inexhaustible, seawater desalination technology plays a crucial role in obtaining fresh water [3]. Seawater desalination is a technology that produces fresh water from non-fresh sources, which has become a vital approach for coastal countries and regions to alleviate freshwater scarcity [4]. Therefore, developing a new, efficient, energy-saving, environmentally friendly, and easy-to-operate water treatment technology has become an urgent need. Capacitive deionization (CDI) technology has emerged in this context and has gained widespread attention [3,5].

CDI is a novel water treatment technology based on the theory of electrochemical double-layer capacitors. When an external DC voltage is applied between two porous electrodes, an electrostatic field forms on the electrode surfaces [6]. Under the influence of this electric field force, cations move towards the cathode and anions towards the anode, being adsorbed within the electric double layers on the electrode surfaces, thereby reducing the ion concentration in the bulk solution and achieving water purification [3,5]. When the electrodes become saturated, they can be regenerated by short-circuiting or applying a reverse voltage [1,7]. This reversible adsorption–desorption process endows CDI technology with excellent cyclic operation performance [5,8]. Despite these advantages, the presence of co-ion repulsion in CDI reduces charge efficiency, leading to low salt adsorption capacity [9]. To overcome the inherent “co-ion effect” within porous carbon electrodes, membrane capacitive deionization (MCDI), which applies ion exchange membranes (IEMs) on the electrode surfaces, has attracted increasing attention [10]. MCDI, due to its IEMs, can effectively inhibit the adsorption of ions with the same charge as the electrode during the adsorption stage and prevent desorbed ions from being re-adsorbed onto the opposite electrode during the regeneration stage, thus avoiding secondary contamination and significantly enhancing adsorption efficiency and regeneration performance [11], enabling more efficient desalination.

Electrode materials are the core of CDI technology, and their performance directly determines desalination efficiency, adsorption capacity, and cyclic stability [12]. Carbon materials are widely studied due to their advantageous properties such as high specific surface area, good electrical conductivity, and chemical stability [13,14]. However, a major challenge for graphene in CDI applications is the tendency for its sheets to agglomerate and stack. Due to strong van der Waals forces and π-π interactions between graphene sheets, irreversible agglomeration easily occurs during preparation, resulting in a practically usable specific surface area far lower than the theoretical value [3,15]. This agglomeration not only reduces the specific surface area but also blocks ion transport channels, affecting the ion transport rate of the electrode material [16]. To address this issue, heteroatom doping introduces more defect sites into the carbon lattice and induces charge redistribution, which significantly enhances the electrochemical performance of pure carbon materials [17]. Kou et al. prepared trace cobalt, nitrogen co-doped porous graphene (CoNG) via an exfoliation–evaporation strategy, achieving a high desalination capacity of 69.23 mg g^−1^ and a fast desalination rate of 2.307 mg g^−1^ min^−1^ in an MCDI system [18]. As suggested by the DFT calculations and XANES/EXAFS results in a previous study on a similar Co-N co-doped graphene system [18], the possible Co-N coordination structure may play a key role in reducing adsorption energy and enhancing electrochemical stability. Xu et al. composited a p-type covalent organic framework (TAPA-COF) with graphene, utilizing interfacial π-π interactions to achieve efficient charge redistribution, resulting in an ultra-high salt adsorption capacity of 88.1 mg g^−1^ in a hybrid capacitive deionization (HCDI) system [19]. Despite these advances, materials based on heteroatom doping still require further improvement, and the synergistic effects of Co-induced graphitization and Zn-assisted pore generation in electrospun carbon nanofibers for MCDI remain largely unexplored.

This study aims to prepare a CoNG@V@CNF material using bimetallic Co/Zn-ZIF-L as a precursor via electrospinning combined with high-temperature carbonization. Notably, “bimetallic” refers to the precursor composition, as Zn acts as a sacrificial template and largely volatilizes during carbonization, while Co remains in the final CoNG@V@CNF electrode. Unlike previous reports that often rely on low-yield or batch-limited synthesis methods (e.g., exfoliation–evaporation or MOF-derived carbons with limited scalability), our electrospinning-based approach offers a high yield, continuous production, and excellent reproducibility, making it a promising candidate for scalable MCDI electrode fabrication. This work therefore bridges the gap between high-performance material design and practical industrial application. The as-prepared CoNG@V@CNF is employed as an electrode material for MCDI systems. The introduction of trace Co nanoparticles can enhance the stability of porous graphene. Meanwhile, N doping contributes to the improved wettability and electronic conductivity of the carbon material. The one-dimensional fiber structure facilitates the construction of a three-dimensional conductive network and provides rapid ion transport channels. Furthermore, this study achieved a high salt adsorption capacity of 58.28 mg g^−1^ and an ultra-fast rate performance of 1.94 mg g^−1^ min^−1^, offering a new design strategy for developing high-performance CDI electrode materials.

## 2. Method

### 2.1. Chemical Reagents

The 2-methylimidazole (2-MIM), Co(AC)_2_·4H_2_O, N-methylpyrrolidone (NMP), polyacrylonitrile (PAN), polystyrene (PS), and N,N-dimethylformamide (DMF) used in this study were purchased from Shanghai Aladdin Biochemical Technology Co., Ltd (Shanghai, China). Polyvinylidene fluoride (PVDF), carbon black (CB), and Zn(AC)_2_ were purchased from Shanghai Titan Technology Co., Ltd. (Shanghai, China).

### 2.2. Preparation of Co/Zn-ZIF-L and Zn-ZIF-L Materials

The material synthesis was carried out following the method described by Fang et al. [20] with minor modifications. In a standard synthesis procedure, first, 2.6 g of 2-methylimidazole (2-MIM) was uniformly dispersed in 80 mL of deionized (DI) water to obtain solution A. A total of 0.498 g of cobalt acetate tetrahydrate (Co(AC)_2_·4H_2_O) and 0.367 g of zinc acetate (Zn(AC)_2_) were dissolved in 80 mL of DI water to obtain solution B, corresponding to a Co:Zn molar ratio of 1:1. Subsequently, under stirring conditions at room temperature, solution B was added dropwise to solution A and stirred continuously for 4 h. After the reaction, the product was collected by centrifugation, washed with DI water 2–3 times, and dried overnight in an oven at 80 °C to obtain the solid Co/Zn-ZIF-L product. The preparation method for Zn-ZIF-L was the same as above, except that solution B was 0.734 g of zinc acetate (Zn(Ac)_2_) dissolved in 80 mL of DI water.

### 2.3. Preparation of CoNG@V@CNF Material

First, 500 mg of polyacrylonitrile (PAN), 300 mg of polystyrene (PS), and 50 mg of the prepared Co/Zn-ZIF-L material were mixed. Subsequently, the mixture was dissolved in 10 mL of N,N-dimethylformamide (DMF) with stirring to ensure complete dissolution, and stirred continuously at 70 °C for 12 h to obtain a homogeneous spinning solution. The obtained uniform solution was loaded into a 5 mL syringe, and the syringe was installed into an electrospinning apparatus. An electric field of 14 kV was applied, and the distance between the cathode and anode was set to be 20 cm. Throughout the electrospinning process, a flow rate of 0.5 mL h^−1^ was used. Subsequently, the obtained composite nanofibers were heated at 200 °C for 3 h in an oven for stabilization treatment. Finally, the stabilized fibers were annealed and carbonized at 800 °C under Ar atmosphere with a ramp rate of 10 °C min^−1^ to obtain the final material, CoNG@V@CNF. The furnace was then cooled to 100 °C at a rate of 10 °C min^−1^.

### 2.4. Electrochemical Measurements

The prepared active material, polyvinylidene fluoride (PVDF), and carbon black (CB) were weighed in a mass ratio of 8:1:1, mixed, and dispersed in an appropriate amount of N-methylpyrrolidone (NMP) with thorough stirring to form a uniform slurry. The resulting slurry was uniformly coated onto graphite paper, ensuring a mass loading of 1.0 ± 0.2 mg cm^−2^. The coated electrode was dried overnight in a vacuum oven at 80 °C. Electrochemical tests were performed using a DH7006 electrochemical workstation with a three-electrode system, where the prepared electrode served as the working electrode, a platinum sheet as the counter electrode, and Ag/AgCl as the reference electrode. Test methods included cyclic voltammetry (CV), galvanostatic charge–discharge (GCD), and electrochemical impedance spectroscopy (EIS).

### 2.5. Desalination Performance Testing

Electrodes for the MCDI system were prepared using the same formulation and process described in Section 2.4. The size of a single electrode was 35 mm × 35 mm, and the total mass of active material on both electrodes was approximately 15 mg (7.5 mg per electrode). All SAC values were normalized to this total mass. Heterogeneous ion exchange membranes (model HMTECH-1680, Hangzhou Huamo Technology Co., Ltd., Hangzhou, China) were used as the anion and cation exchange membranes. Prior to use, the membranes were gently wiped with ethanol to remove surface contaminants and then immersed in deionized water overnight (≥12 h) to ensure complete hydration and removal of soluble impurities. The MCDI cell was assembled in a sandwich configuration following the sequence: acrylic end plate→electrode sheet→cation exchange membrane→central spacer frame→anion exchange membrane→electrode sheet→ acrylic end plate. The entire cell was clamped tightly by screws. The feed solution was 40 mL of NaCl aqueous solution, with the initial concentration adjusted within the range of 500–3000 ppm. Driven by a peristaltic pump, the solution circulated through the MCDI cell at a flow rate of 40 mL min^−1^. Each adsorption and desorption stage was conducted for 30 min, with desorption performed by applying a reverse voltage of −1.4 V for 30 min. Under a constant temperature of 298 K, a conductivity meter (DDSJ-308F, Leici, INESA Scientific Instrument Co., Ltd., Shanghai, China) was used to continuously record the conductivity of the solution. The conductivity values were converted to NaCl concentrations using a pre-established calibration curve (Figure S6). The curve shows excellent linearity (R^2^ = 0.9997) over the concentration range used in this study, and was confirmed to be consistent across all three electrode materials.

To scientifically and objectively evaluate the performance of the capacitive deionization system, a series of quantitative indicators are typically employed, the most important of which include salt adsorption capacity, desalination rate, charge efficiency, and energy consumption.

Salt Adsorption Capacity (SAC) refers to the mass of salt adsorbed per unit mass of electrode material at saturation and is a core indicator for evaluating the desalination capability of electrode materials. Its calculation formula is as follows:

(1) $$SAC=\frac{(C_{0}-C_{1})V}{M}$$

where C_0_ and C_1_ represent the initial and final salt concentrations (mg L^−1^) in the electrolyte, respectively. V is the volume of the electrolyte (L), and M is the total mass of the electrode. High specific surface area and a reasonable pore size distribution are key to achieving high SAC.

Desalination Rate (SAR) is usually expressed as the average salt adsorption rate, i.e., the average amount adsorbed per unit mass of electrode material per unit time, reflecting the adsorption kinetics of the electrode material. Its calculation formula is as follows:

(2) $$SAR=\frac{{SAC}_{t}}{t}$$

where SAC_t_ is the desalination capacity of the CDI device over a cycle time t, and t is the total time for one complete cycle of the CDI device for desalination.

Charge efficiency (Λ) reflects the efficiency of the electrode material in utilizing charge. The calculation formula is as follows:

(3) $$\Lambda =\frac{\Gamma \times Ϝ}{\Sigma }$$

where Γ is the SAC (mol g^−1^), F is the Faraday constant, and Σ is integrated charge (C g^−1^). High charge efficiency implies less energy loss and is an important parameter for measuring the energy utilization efficiency of a CDI system.

Energy consumption (E, Wh g^−1^) is calculated using the following formula:

(4) $$E=\frac{\int_{0}^{t}V_{cell}\times I}{C\times V_{soln}}$$

where V_cell_ is the operating voltage (V) during the CDI desalination process at time t, I is the current (A) at time t, C is the difference in electrolyte concentration before and after CDI desalination (g L^−1^), and V_soln_ is the total volume (L) of the NaCl electrolyte in the CDI desalination system.

## 3. Results and Discussion

### 3.1. Synthesis and Morphology of Electrode Materials

#### Morphology and Phase Analysis

**Figure 1:**
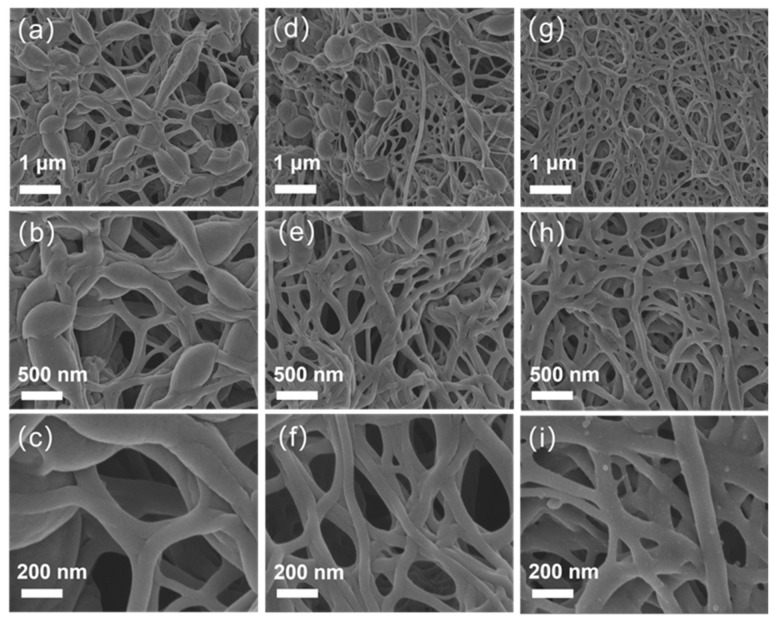
(**a**–**c**) SEM images of Co@CNF. (**d**–**f**) SEM images of V@CNF. (**g**–**i**) SEM images of CoNG@V@CNF.

The surface morphology of Co@CNF, V@CNF and CoNG@V@CNF was observed by scanning electron microscopy (SEM). SEM images of Co@CNF are shown in Figure 1a–c. It can be observed from Figure 1a that the fibers are unevenly distributed, with obvious beads, and the fibers are intertwined to form a dense network structure. As can be noticed from Figure 1b,c, the fiber surface has no obvious granular features and the fibers are continuous. Compared with Co@CNF, V@CNF exhibits different distribution characteristics at 1 μm scale (Figure 1d), with a denser fiber network and fewer beads. Figure 1e,f further indicate that the fiber diameter is close to that of Co@CNF fibers, and there are no obvious nano-scale protrusions on the fiber surface. Figure 1g–i show SEM images of CoNG@V@CNF. The fiber network structure more uniform characteristics with only a few beads. Figure 1h,i show new morphological features on the fiber surface, and the fiber diameter is thinner than that of Co@CNF and V@CNF. This surface feature only occurs in bimetallic systems and may be related to the co-introduction of Zn^2+^ and Co^2+^ [21,22]. Although bead formation in Co@CNF may reduce mechanical strength and surface area, the bimetallic CoNG@V@CNF exhibits more uniform fibers and superior performance due to pseudocapacitive contributions from Co-related species and Zn-derived porosity.

**Figure 2:**
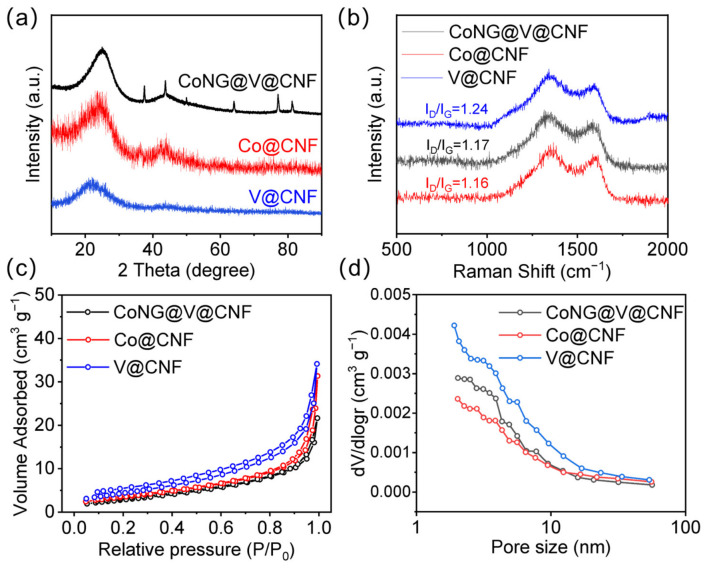
(**a**) XRD patterns of CoNG@V@CNF, Co@CNF, and V@CNF. (**b**) Raman spectra of CoNG@V@CNF, Co@CNF, and V@CNF. (**c**) Nitrogen adsorption–desorption isotherms of CoNG@V@CNF, Co@CNF, and V@CNF. (**d**) Pore size distribution curves of CoNG@V@CNF, Co@CNF, and V@CNF.

As shown in Figure 2a, the X-ray diffraction (XRD) patterns of CoNG@V@CNF, Co@CNF, and V@CNF all exhibit a broad diffraction peak centered around 24°, corresponding to the (002) plane of carbon materials. This indicates the successful formation of a carbon fiber framework after electrospinning, followed by high-temperature carbonization. In addition to the peak at 24.99°, CoNG@V@CNF shows several distinct diffraction peaks at 37.59°, 43.68°, 50.00°, 64.02°, and 77.06°. Among them, the peaks at 43.68°, 50.00°, and 77.06° can be attributed to crystalline Co species, while those at 37.59° and 64.02° are likely associated with cobalt oxides, indicating the formation of a hybrid active phase comprising metallic Co, Co_3_O_4_, and CoO after carbonization of the bimetallic precursor. The XRD pattern of CoNG@V@CNF shows diffraction peaks corresponding to crystalline Co species. Based on the SEM images (Figure 1g–i), the fiber surface appears smooth with no visible nanoparticles, indicating that the Co nanoparticles are embedded within the carbon matrix rather than aggregated on the surface. In contrast, Co@CNF only exhibits weak peaks around 36° and 43°, suggesting a lower crystallinity or limited loading of Co species. For V@CNF, no obvious Zn-related diffraction peaks are observed except for the carbon peak at 24°, implying that Zn is volatilized during high-temperature carbonization and acts as a sacrificial template to promote pore formation. Compared with the monometallic systems, CoNG@V@CNF possesses more abundant crystalline phases and a higher content of active species, which is beneficial for enhancing electrical conductivity and electrochemical performance.

Figure 2b presents the Raman spectra of the three samples. All samples exhibit two characteristic peaks at approximately 1350 cm^−1^ and 1580 cm^−1^, corresponding to the D band and G band, respectively. The D band is associated with defects, edge sites, and structural disorder in carbon materials, while the G band represents graphitic sp^2^ hybridized carbon. Among them, V@CNF exhibits the highest I_D_/I_G_ value of 1.24, indicating the most defect sites and the lowest graphitization degree. Co@CNF demonstrates the lowest I_D_/I_G_ value of 1.16, reflecting its highest graphitization degree. CoNG@V@CNF has an I_D_/I_G_ value of 1.17, intermediate between the two, indicating a well-developed graphitic structure while preserving certain defects.

To further verify the surface functional groups and bonding configurations of the obtained materials, the FTIR spectra have been collected. As shown in Figure S1, the characteristic band located at approximately 1580 cm^−1^ can be assigned to C=N/C=C stretching vibrations, indicating the successful incorporation of nitrogen-containing species into the carbon framework. The detailed FTIR peak assignments, including the corresponding functional groups and literature references, are summarized in Table S1 [23]. Meanwhile, the clear blue shift in the C=N band (from 1562.94 cm^−1^ in V@CNF to 1603.96 cm^−1^ in CoNG@V@CNF) suggests possible interactions between Co and N species. The abundant surface functional groups on CoNG@V@CNF are also beneficial for improving hydrophilicity and providing ion-accessible active sites.

The N_2_ adsorption–desorption isotherms shown in Figure 2c display typical Type IV behavior with a sharp adsorption increase at high relative pressure (P/P_0_ > 0.9), suggesting the coexistence of mesopores and macropore-like voids generated during carbonization. In the low-pressure region (P/P0 < 0.1), which primarily reflects micropore filling, V@CNF exhibits a distinctly higher adsorption capacity, whereas CoNG@V@CNF and Co@CNF show lower capacities, consistent with their respective specific surface areas. Among the three materials, V@CNF possesses the highest specific surface area at 16.65 m^2^ g^−1^, followed by Co@CNF at 10.69 m^2^ g^−1^, while CoNG@V@CNF has the lowest specific surface area of only 11.06 m^2^ g^−1^. The pore size distribution curves in Figure 2d further confirm that CoNG@V@CNF possesses a hierarchical porous structure dominated by small mesopores (~2 nm), with a minor microporous contribution. Such a mesopore-dominant structure is favorable for capacitive deionization, as mesopores serve as ion-buffering reservoirs and fast diffusion channels, while the limited micropores provide additional ion electrosorption sites. Compared with Co@CNF and V@CNF, although CoNG@V@CNF exhibits a relatively low specific surface area (11.06 m^2^ g^−1^) compared to conventional porous carbons, it presents a well-defined mesoporous structure with an interconnected pore network. Importantly, the excellent electrochemical performance of CoNG@V@CNF is primarily governed by pseudocapacitive contributions from Co-related species (Co_3_O_4_/CoO), as confirmed by the scan-rate-dependent CV analysis (Figure S3), where the diffusion-controlled contribution reaches 90.03% at 1 mV s^−1^. Therefore, the relatively low surface area is not a limiting factor, as the pseudocapacitive mechanism does not rely on a high surface area. The mesoporous structure mainly facilitates ion transport, while the carbon framework serves as a conductive scaffold for the Co nanoparticles. This synergistic effect of Zn evaporation-induced pore generation and Co-assisted graphitization during pyrolysis results in a rationally engineered pore architecture that simultaneously promotes ion transport, charge storage, and adsorption kinetics.

### 3.2. Electrochemical Performance

**Figure 3:**
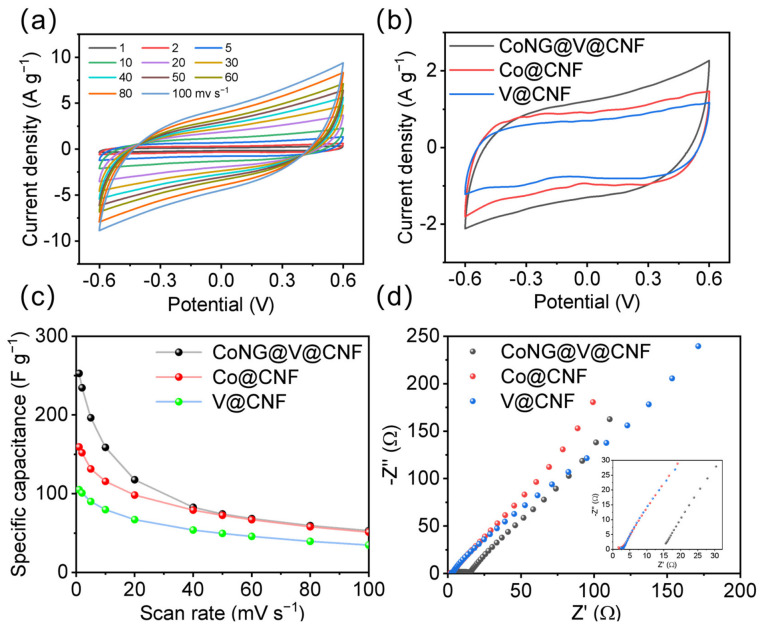
(**a**) CV curves of the CoNG@V@CNF electrode at different scan rates. (**b**) Comparison of CV curves of CoNG@V@CNF, Co@CNF, and V@CNF at a scan rate of 10 mV s^−1^. (**c**) Comparison of the specific capacitance of CoNG@V@CNF, Co@CNF, and V@CNF electrodes at different scan rates. (**d**) Nyquist plots of CoNG@V@CNF, Co@CNF, and V@CNF electrodes; the inset shows the enlarged high-frequency region of the Nyquist plots.

To evaluate the energy storage capability and charge transport properties of the three materials as CDI electrodes, cyclic voltammetry (CV), and electrochemical impedance spectroscopy (EIS), measurements have been conducted. Figure 3a presents the CV curves of the CoNG@V@CNF electrode at different scan rates (1–100 mV s^−1^). As shown, all CV curves exhibit a nearly rectangular shape without obvious redox peaks across the entire potential window (−0.6 to 0.6 V), indicating that the capacitive behavior is dominated by an electric double layer. Co@CNF and V@CNF also exhibit nearly rectangular CV curves across the same potential window of −0.6 to 0.6 V, as indicated in Figure S2, indicating that electric double-layer capacitance is the dominant charge storage mechanism for all three materials. Even at a high scan rate of 100 mV s^−1^, the CV curves remain well-shaped without significant distortion, confirming the fast ion transport kinetics and low charge transfer resistance of the CoNG@V@CNF electrode. Figure 3b compares the CV curves of the three materials at the same scan rate of 10 mV s^−1^. CoNG@V@CNF exhibits the largest enclosed area, indicating the highest charge storage capacity. This can be attributed to its hierarchical porous structure, which provides a larger electrolyte-accessible surface area, while Co nanoparticles and nitrogen doping enhance electrical conductivity, increase active surface sites, and improve surface wettability. Figure 3c shows the variation in specific capacitance for the three samples at different scan rates. The specific capacitance of all electrodes decreases with an increasing scan rate, which is due to the insufficient time for electrolyte ions to fully penetrate into the internal pores at high scan rates. At a low scan rate of 1 mV s^−1^, the specific capacitances of CoNG@V@CNF, Co@CNF, and V@CNF reach approximately 252.76 F g^−1^, 159.28 F g^−1^, and 105.09 F g^−1^, respectively. To understand the origin of this high capacitance, we quantified the capacitive and diffusion-controlled contributions using scan-rate-dependent CV analysis in Figure S3. At 1 mV s^−1^, the diffusion-controlled contribution reaches 90.03%, indicating that pseudocapacitive processes from Co-related species (Co_3_O_4_/CoO) dominate the charge storage at low scan rates. As the scan rate increases to 100 mV s^−1^, the diffusion-controlled contribution decreases to 52.48%, while the capacitive contribution increases to 47.52%, consistent with the kinetic limitation of faradaic reactions at higher scan rates. Even at 100 mV s^−1^, CoNG@V@CNF maintains a capacitance of 53 F g^−1^, demonstrating excellent rate performance. These results further confirm that the bimetal-derived structure significantly enhances the capacitive performance of the electrode.

Electrochemical impedance spectroscopy (EIS) is used to evaluate the conductivity of the electrodes after ion adsorption, as shown in Figure 3d, with the inset illustrating the magnified high-frequency region. All three materials exhibit small semicircles in the high-frequency region, indicating low charge transfer impedance and excellent interfacial charge transport capabilities. The intercept values in the high-frequency range reveal that the Co@CNF electrode demonstrates the lowest initial value along the Z axis, reflecting its minimum solution resistance (Rs) and superior conductivity, followed by V@CNF, while CoNG@V@CNF exhibits relatively higher Rs (Rs_Co@CNF_ = 1.66 Ω, Rs_V@CNF_ = 2.38 Ω, and Rs_CoNG@V@CNF_ = 15.56 Ω). In the low-frequency region, the Co@CNF electrode displays a nearly vertical linear characteristic, indicating superior capacitive behavior and faster ion diffusion rates. In contrast, CoNG@V@CNF shows slightly lower diffusion performance but still outperforms V@CNF. Although CoNG@V@CNF lacks an advantage in impedance performance, its hierarchical pore structure, abundant active sites, and excellent electrochemical properties confer higher salt adsorption capacity during capacitive deionization. These results demonstrate that CDI performance depends not only on conductive characteristics but also closely correlates with pore architecture and surface chemical properties.

To further evaluate the rate capability under constant current conditions, galvanostatic charge–discharge (GCD) measurements are performed, and the results are presented in Figure S4. All three electrodes exhibit nearly symmetric triangular-shaped GCD curves, indicating good electrochemical reversibility. However, it is noteworthy that while CoNG@V@CNF and V@CNF can be stably cycled within a potential window of −0.6 to 0.6 V (Figure S4a,b), the GCD testing of Co@CNF is limited to a narrower range of −0.2 to 0.6 V (Figure S4c), as stable charge–discharge behavior cannot be obtained when extending to −0.6 V. This suggests that the monometallic Co-derived carbon suffers from poor electrochemical stability under constant current polarization at more negative potentials, a limitation that is effectively mitigated in the bimetallic Co/Zn-derived CoNG@V@CNF system. Among the three electrodes, CoNG@V@CNF displays the longest charge/discharge duration at any given current density, consistent with its highest specific capacitance. Even at high current densities, its GCD curves maintain good symmetry without a significant IR drop, confirming the excellent rate capability suggested by the CV analysis.

### 3.3. Desalination Performance

**Figure 4:**
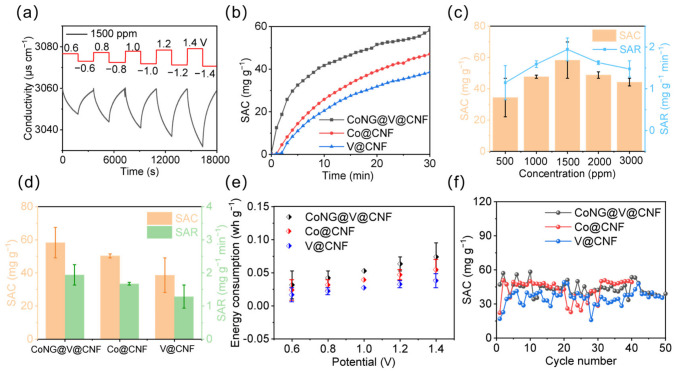
(**a**) Conductivity transients of CoNG@V@CNF at different voltages with a 1500 ppm feed solution. (**b**) SAC of CoNG@V@CNF, Co@CNF, and V@CNF in a 1500 ppm feed solution at an operating voltage of 1.4 V. (**c**) SAC and SAR of the CoNG@V@CNF electrode in an MCDI system at an operating voltage of 1.4 V under different feed concentrations. (**d**) SAC and SAR of CoNG@V@CNF, Co@CNF, and V@CNF in an MCDI system using a 1500 ppm feed solution at 1.4 V. (**e**) Comparison of energy consumption among CoNG@V@CNF, Co@CNF, and V@CNF. (**f**) Cycling stability of CoNG@V@CNF, Co@CNF, and V@CNF in an MCDI system using a 1500 ppm feed solution at 1.4 V.

The relationship between applied voltage and desalination performance is first evaluated using a 1500 ppm NaCl solution, as shown in Figure 4a. As the applied voltage increases from 0.6 V to 1.4 V, the decrease in solution conductivity becomes more pronounced, indicating enhanced desalination capability, with optimal performance achieved at 1.4 V. Therefore, 1.4 V was selected as the operating voltage for subsequent experiments. This voltage-dependent behavior is further verified across a broad concentration range of 500 to 3000 ppm, as presented in Figure S5. At all tested concentrations, higher applied voltages lead to a more significant conductivity drop during the adsorption stage, i.e., better desalination performance, which is consistent with electric double-layer theory. At lower concentrations (500 and 1000 ppm), the conductivity decay curves are smooth and stable, indicating that ion transport was not rate-limiting. The decline in desalination performance at higher concentrations (2000–3000 ppm) can be attributed to concentration polarization and partial pore blockage. Although ion exchange membranes can mitigate concentration polarization, they do not fully eliminate it, mainly due to increased membrane resistance and severe diffusion limitations at high ionic strengths, which hinder ion transport even in the presence of membranes. Additionally, the high ion flux can cause localized pore blockage within the carbon electrode. Notably, upon applying a reverse voltage, the conductivity rapidly returns to its initial level across all concentrations, demonstrating the excellent regeneration capability of the CoNG@V@CNF electrode-based CDI system. Figure 4b shows the time-dependent adsorption performance of different electrode materials toward a 1500 ppm NaCl solution at 1.4 V. It can be clearly observed that the CoNG@V@CNF electrode exhibits the highest adsorption capacity. Due to Zn volatilization during high-temperature carbonization, abundant micropores are formed, resulting in a relatively high specific surface area for Zn-ZIF-L-derived carbon. However, its limited graphitization degree leads to restricted ion transport kinetics. In contrast, Co-ZIF-L-derived carbon benefits from Co-catalyzed graphitization, which improves electronic conductivity, but its less developed pore structure limits further enhancement in adsorption capacity. Compared with these monometallic systems, the Co/Zn bimetallic system exhibits a pronounced synergistic effect. Zn volatilization constructs abundant micropores, Co promotes graphitization and enhances conductivity, and N doping increases surface polarity and active sites. As a result, the Co/N-doped porous carbon fiber possesses a high surface area, excellent conductivity, hierarchical pore structure, and abundant electrochemical active sites. To investigate the desalination performance of different electrode materials in the MCDI system, intermittent CDI measurements were conducted in NaCl solutions with varying concentrations under different feed concentrations ranging from 500 ppm to 3000 ppm. As shown in Figure 4c, the salt adsorption capacity (SAC) and salt adsorption rate (SAR) of the CoNG@V@CNF electrode are evaluated. With the increase in initial salt concentration from 500 ppm to 1500 ppm, the SAC shows a significant upward trend and reaches a maximum value of 58.28 mg g^−1^ at 1500 ppm. The experiment with 58.28 mg g^−1^ exceeds the theoretical EDL-predicted value of approximately 0.37 mmol g^−1^, suggesting additional pseudocapacitive contributions from Co-related species (Co_3_O_4_/CoO). This is consistent with previous reports on cobalt oxide-based desalination systems, where faradaic reactions enhance ion storage beyond pure EDL mechanisms [9,18]. The high specific capacitance at low scan rates further supports that both EDL and pseudocapacitive processes contribute to the total charge storage. When the concentration further increases to 2000–3000 ppm, the SAC slightly decreases, and the SAR exhibits a similar trend. This behavior can be explained in terms of ion transport and electric double-layer (EDL) formation mechanisms. At low salt concentrations, the limited number of ions in the solution leads to insufficient EDL formation on the electrode surface, and the micropores are not fully utilized, resulting in relatively low adsorption capacity. When the concentration increases to 1500 ppm, the ionic strength of the solution is enhanced, the ion diffusion driving force increases, and the hierarchical porous structure of the electrode is fully wetted, enabling efficient EDL formation. However, at higher concentrations, although the number of ions continues to increase, concentration polarization becomes more severe and partial pore blockage may occur, leading to a slight decrease in SAC. In addition, ion crowding in micropores at high concentrations may further limit the accessible adsorption sites, as the limited pore volume becomes saturated and hinders further ion adsorption. This result indicates that the hierarchical porous carbon fiber derived from Co/Zn-ZIF-L via electrospinning achieves optimal structure–performance matching under moderate salt concentration conditions.

Figure 4d compares the SAC and SAR of the three electrode materials in a 1500 ppm NaCl solution at 1.4 V. CoNG@V@CNF exhibits significantly higher SAC and SAR than Co@CNF and V@CNF, demonstrating a superior desalination performance and faster adsorption kinetics. This enhancement is attributed to the synergistic effect of Co/Zn bimetallic-derived hierarchical porosity, a well-established conductive network, and abundant surface active sites, which collectively improve ion transport efficiency and charge storage capability. To contextualize the desalination kinetics of our CoNG@V@CNF electrode within the broader literature, we compared its performance against representative state-of-the-art carbon-based CDI materials. As summarized in Table S2 [24,25,26,27,28,29,30,31], several advanced carbon electrodes exhibit SAR values in the range of 2–5 mg g^−1^ min^−1^. However, such high rates are often achieved at the expense of a relatively low SAC (typically 20–40 mg g^−1^). In contrast, our electrode achieves a competitive SAR of 1.94 mg g^−1^ min^−1^ while maintaining a high SAC of 58.28 mg g^−1^, indicating a favorable balance between adsorption capacity and rate. This comparison further supports the view that the desalination performance of CoNG@V@CNF is competitive among recently reported carbon-based MCDI systems.

Figure 4e presents the specific energy consumption of the three electrodes at different operating voltages. The energy consumption of all samples increases with increasing applied voltage, indicating that although higher voltage enhances ion migration and adsorption, it also increases energy input and interfacial losses. Under the same voltage conditions, CoNG@V@CNF exhibits higher energy consumption than Co@CNF and V@CNF, reaching approximately 0.074 Wh g^−1^ at 1.4 V. In contrast, V@CNF shows the lowest energy consumption, but its desalination performance is also inferior. Therefore, low energy consumption alone does not necessarily indicate superior material performance. A comprehensive evaluation should consider both desalination capacity and adsorption efficiency. The charge efficiency (Λ) of the CoNG@V@CNF electrode was calculated using Equation (3). At an operating voltage of 1.4 V in 1500 ppm NaCl solution, Λ ranges from 62.58% to 95.88% over 10 cycles, with an average value of approximately 80.72% (Table S3). This high charge efficiency indicates that the majority of the charge passed during the adsorption step is effectively utilized for salt adsorption, which is consistent with the favorable pore structure and pseudocapacitive contributions from Co-related species of the CoNG@V@CNF electrode. Figure 4f shows the cycling stability of different electrode materials in a 1500 ppm NaCl solution at 1.4 V. For CoNG@V@CNF, the SAC decreases from 47.1 mg g^−1^ in the first cycle to approximately 39.1 mg g^−1^ after 50 adsorption–desorption cycles, corresponding to a capacity retention of ~83%. The majority of the capacity loss occurs within the first 20 cycles, after which the decay rate slows considerably. This initial fading may be attributed to the gradual activation and stabilization of the electrode surface, as well as minor structural rearrangement during repeated cycling. In comparison, V@CNF exhibits fluctuating SAC values without a clear stabilization trend over 50 cycles, while Co@CNF shows comparable SAC values to CoNG@V@CNF in the later cycles but with lower initial capacity. These results indicate that CoNG@V@CNF maintains a competitive SAC and relatively stable performance over the tested 50 cycles. However, we acknowledge that the observed capacity fading, particularly after 40 cycles, indicates room for further optimization of the long-term structural stability. The initial cycling stability of CoNG@V@CNF is supported by the highly graphitized carbon framework and the encapsulation of Co nanoparticles within the carbon layer, which help mitigate aggregation during the early cycles. In our future work, we will conduct longer cycling tests (e.g., 100+ cycles) and post-cycling structural/compositional characterization to fully evaluate the practical viability of this material.

## 4. Conclusions

This paper systematically investigates the preparation method, structural regulation, and CDI performance of a CoNG@V@CNF electrode constructed using Co/Zn-ZIF-L as a precursor via electrospinning combined with high-temperature carbonization. Furthermore, unlike many previously reported high-performance CDI materials that suffer from low synthesis yield or poor scalability, our electrospinning-based fabrication strategy enables high-yield, continuous production, positioning CoNG@V@CNF as a competitive candidate for future industrial-scale MCDI applications. The research focuses on exploring and understanding the influence mechanisms of Co and N doping on the microstructure, electrochemical behavior, and desalination performance of the carbon fiber material. Through systematic structural characterization and performance comparison with control materials, which is Co@CNF and V@CNF, this paper deeply analyzes the effects of mono-metallic and bimetallic precursor systems on the characteristics of derived carbon materials. The differences in morphology, specific capacitance, impedance behavior, and salt adsorption capacity among the three electrode materials are systematically evaluated. Compared to traditional carbon-based electrode materials, the CoNG@V@CNF composite exhibits promising advantages, primarily attributed to its unique chemical and structural characteristics. The doped Co species enhance the stability of porous graphene, N doping modulates the electronic structure and introduces active sites, and the one-dimensional fiber structure forms a continuous conductive network. Under conditions of 1500 ppm and 1.4 V, the CoNG@V@CNF achieves a maximum SAC of 58.28 mg g^−1^ and a SAR of 1.94 mg g^−1^ min^−1^. It should be noted that the current study was conducted at the laboratory scale with a single-cell configuration. Further long-term stability tests (e.g., 100+ cycles), scale-up studies, and continuous-flow operation evaluations are necessary before the practical implementation of this electrode material. This work provides a new technical pathway for the structural design of high-performance CDI electrode materials.

## Data Availability

The original contributions presented in this study are included in the article/Supplementary Material. Further inquiries can be directed to the corresponding authors.

## Supplementary Materials

The following supporting information can be downloaded at: https://www.mdpi.com/article/10.3390/membranes16070243/s1, Figure S1: FTIR spectra of CoNG@V@CNF, Co@CNF, and V@CNF. Figure S2: (a) CV curves of the Co@CNF electrode at different scan rates. (b) CV curves of the V@CNF electrode at different scan rates. Figure S3: Capacitive and diffusion-controlled contribution ratios of CoNG@V@CNF calculated from CV curves at scan rates of 1–100 mV s^−1^. Figure S4: (a–c) Galvanostatic charge-discharge curves of CoNG@V@CNF, Co@CNF, and V@CNF at different current densities. Figure S5: (a) Conductivity transients of CoNG@V@CNF at different voltages with a 500 ppm feed solution. (b) Conductivity transients of CoNG@V@CNF at different voltages with a 1000 ppm feed solution. (c) Conductivity transients of CoNG@V@CNF at different voltages with a 2000 ppm feed solution. (d) Conductivity transients of CoNG@V@CNF at different voltages with a 3000 ppm feed solution. Figure S6: Calibration curve of conductivity versus NaCl concentration for CoNG@V@CNF, Co@CNF, and V@CNF electrodes measured at 298 K. Table S1: FTIR peak assignments and corresponding functional groups for CoNG@V@CNF, Co@CNF, and V@CNF. Table S2: Comparison of desalination performance of representative carbon-based and carbide-derived electrode materials. Table S3: Charge efficiency (Λ) of the CoNG@V@CNF electrode over 10 cycles at 1.4 V in 1500 ppm NaCl solution.
