# Supplementary material for: Boosting Capacitive Deionization Performance via Bimetallic Synergistic Engineering of Electrospun Co/N-Doped Porous Carbon Nanofibers
Source: Membranes (Basel). 2026 Jul 17;16(7):243. doi: 10.3390/membranes16070243 (PMC13414434; doi:10.3390/membranes16070243)
Supplement: Supplementary file 1 [file membranes-16-00243-s001.zip › membranes-4332858-supplementary.pdf]

# Supporting Information

***Boosting Capacitive Deionization Performance via Bimetallic Synergistic Engineering of Electrospun Co/N-Doped Porous Carbon Nanofibers***

*Xinyue Ma [a†], Yuan Li [a†], Kuo Meng [a], Chengbo Kou [a], Binling Li [a], Zhonglei Zhu [a], Haojie Li [a], Zhihan Deng [a], Runze Yang [a], Hupeng Zhou [a], Xin Wang [a], Lang Luo [a], Fuming Chen [b], Chengding Gu [a], Yuxiao Zhang [b*], and Lu Guo [a, b, c*]*

[a] *School of materials and energy, Yunnan University, Kunming 650091, China*

[b] *Yunnan Malaya Institute, School of engineering, Yunnan University, Kunming 650091, China.*

[c] *Yunnan Key Laboratory of Electromagnetic Materials and Devices, School of engineering, Yunnan University, Kunming 650091, China.*

** Corresponding email: yuxiao.zhang@ynu.edu.cn (Y. Z); luguo@ynu.edu.cn (L. G).*

[†] These authors contributed equally to this work.

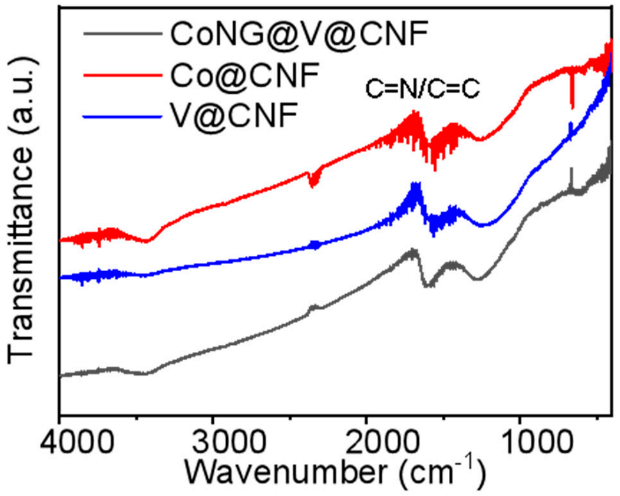


Figure S1. FTIR spectra of CoNG@V@CNF, Co@CNF, and V@CNF.

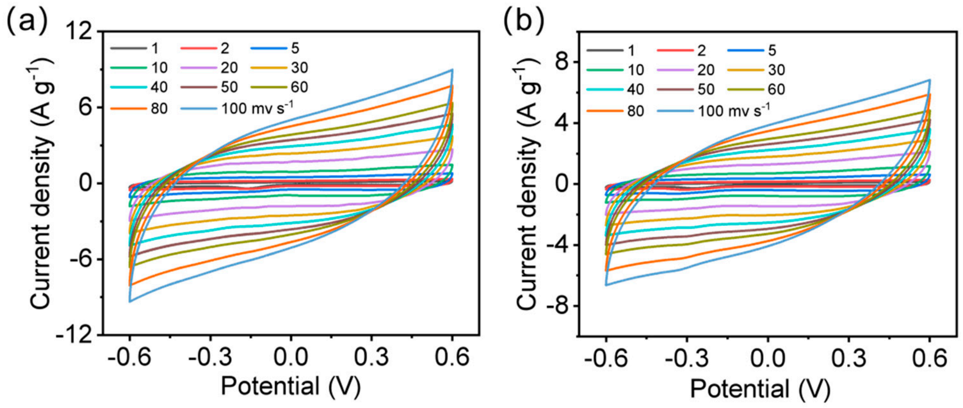


Figure S2. (a) CV curves of the Co@CNF electrode at different scan rates. (b) CV curves of the V@CNF electrode at different scan rates.

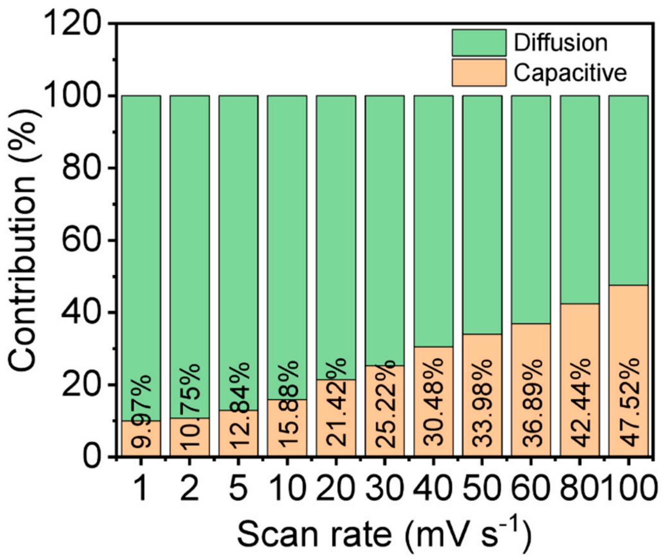


Figure S3. Capacitive and diffusion-controlled contribution ratios of CoNG@V@CNF calculated from CV curves at scan rates of 1-100 mV s$^{-1}$.

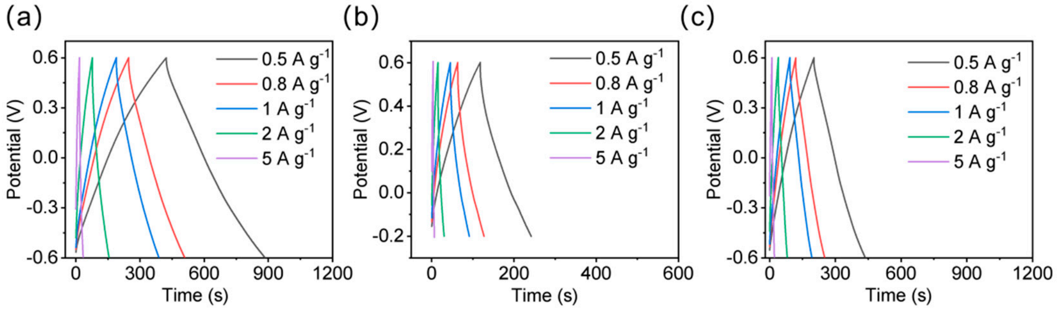


Figure S4. (a-c) Galvanostatic charge-discharge curves of CoNG@V@CNF, Co@CNF, and V@CNF at different current densities.

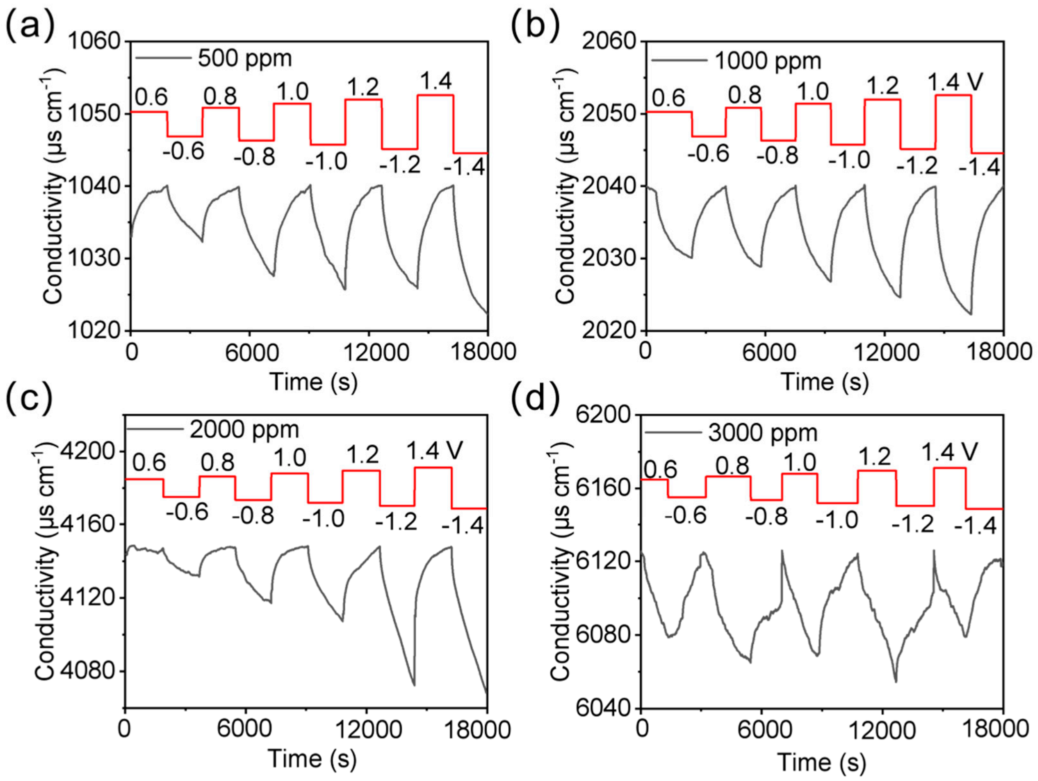


Figure S5. (a) Conductivity transients of CoNG@V@CNF at different voltages with a 500 ppm feed solution. (b) Conductivity transients of CoNG@V@CNF at different voltages with a 1000 ppm feed solution. (c) Conductivity transients of CoNG@V@CNF at different voltages with a 2000 ppm feed solution. (d) Conductivity transients of CoNG@V@CNF at different voltages with a 3000 ppm feed solution.

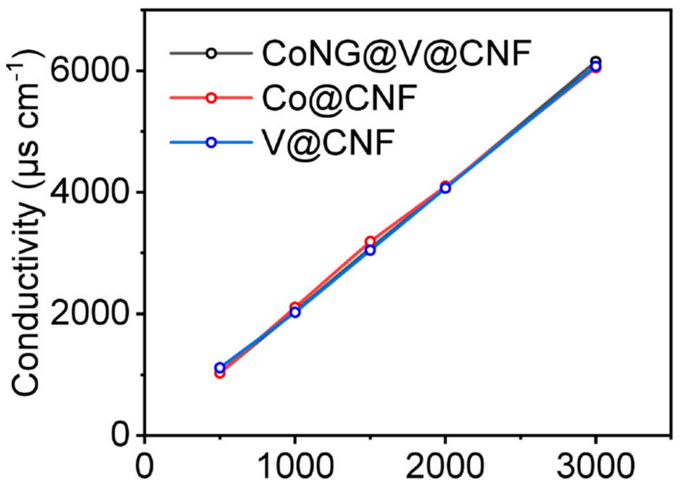


Figure S6. Calibration curve of conductivity versus NaCl concentration for CoNG@V@CNF, Co@CNF, and V@CNF electrodes measured at 298 K

Table S1. FTIR peak assignments and corresponding functional groups for CoNG@V@CNF, Co@CNF, and V@CNF.

| Peak position ($cm^{-1}$) | Functional group | Assignment | Ref. |
|---|---|---|---|
| ~1562-1604 | C=N/C=C | Stretching vibration of aromatic rings and C=N/C=C bonds | |
| ~1525-1530 (blue shift) | C=N (Co-coordinated) | Blue shift indicates possible interaction between Co and N species; metal-N coordination causes blue shift of C=N band | [23] |
| ~3400 | O-H/N-H | Stretching vibration of adsorbed water, surface hydroxyl groups, or N-H | |
| ~2920-2850 | C-H | Aliphatic C-H stretching | |
| ~1620 | C=O/adsorbed $H_2O$ | Carbonyl/carboxyl groups or absorbed water molecules | |

Table S2. Comparison of desalination performance of representative carbon-based and carbide-derived electrode materials

| Material | Voltage (V) | Concentration (ppm) | SAC （mg $g^{-1}$) | SAR (mg $g^{-1}$ | Energy consumption |
|---|---|---|---|---|---|

| | | | | min$^{-1}$) | (kwh kg$^{-1}$) |
|---|---|---|---|---|---|
| biomass-based carbon material [24] | 1.2 | 500 | 41.85 | 17.67 | - |
| CA(K)/CMC [25] | 1.2 | 300 | 17.21 | 1.38 | - |
| AC/GO-15 [26] | 1.0 | 400 | 5.7 | 0.34 | - |
| commercial Activated carbon [26] | 1.0 | 400 | 3.74 | 0.23 | - |
| N, S-doped biochar [27] | 1.2 | 500 | 14.4 | 8.2 | - |
| CoNG-NG [28] | 1.4 | 1000 | 69.23 | 2.307 | 0.36 |
| mPDA/MXene [29] | 1.5 | 1000 | 37.72 | 1.27 | 0.69 |
| $MoS_2$@MXene [30] | 1.2 | 500 | 35.6 | 2.6 | 0.544 |
| $W_{18}O_{49}/Ti_3C_2$ [31] | 1.2 | 500 | 29.25 | 0.97 | 0.5642 |
| Functionalized MXene [32] | 1.2 | 5000 | 49 | 2.92 | 0.38 |
| CoNG@V@CNF | 1.4 | 1500 | 58.28 | 1.94 | 0.074 |

Table S3 Charge efficiency (Λ) of the CoNG@V@CNF electrode over 10 cycles at 1.4 V in 1500 ppm NaCl solution.

| Cycle number | Λ (%) |
|---|---|
| 1 | 76.08% |

| | |
|---|---|
| 2 | 94.76% |
| 3 | 78.43% |
| 4 | 62.58% |
| 5 | 77.82% |
| 6 | 92.44% |
| 7 | 76.08% |
| 8 | 75.16% |
| 9 | 77.94% |
| 10 | 95.88% |

**Reference:**


23. Fanning, P.E.; Vannice, M.A. A DRIFTS study of the formation of surface groups on carbon by oxidation. *Carbon* **1993**, *31*, 721–730.
24. Chen, M.; Liu, W.; Yu, T.; Liu, B.; Wang, M.; Fu, D.; Xue, J.; Wen, H.; Liu, X. Distillers' grains carbon for high-performance capacitive deionization. *Separation and Purification Technology* **2025**, *359*, 130882.
25. Miao, L.; Wang, Z.; Gao, M.; Peng, J.; Chen, Y.; Chen, F.; Chen, W.; Ao, T. A green potassium citrate activation strategy via one-step synthesis of 3D porous carbon for capacitive deionization. *Separation and Purification Technology* **2024**, *346*, 127510.
26. Folaranmi, G.; Bechelany, M.; Sistat, P.; Cretin, M.; Zaviska, F. Comparative Investigation of Activated Carbon Electrode and a Novel Activated Carbon/Graphene Oxide Composite Electrode for an Enhanced Capacitive Deionization. *Materials* **2020**, *13*, 5185.
27. Wang, C.; Li, Y.; Qiao, Y.; Tut, N.L.; Deng, D.; Pan, Q.; Yao, G.; Wang, Y. The regulation of biochar surface potential to achieve rapid capacitive deionization. *Journal of Environmental Chemical Engineering* **2023**, *11*, 111560.
28. Guo, L.; Kou, C.; Fang, D.; Yang, L.; Deng, G.; Zhu, L.; Ji, W.; Zhu, Z.; Li, H.; Meng, K.; et al. Trace cobalt, nitrogen co-doped graphene for high-performance membrane capacitive deionization. *Separation and Purification Technology* **2025**, *367*, 132957.
29. Li, Q.; Xu, X.; Guo, J.; Hill, J.P.; Xu, H.; Xiang, L.; Li, C.; Yamauchi, Y.; Mai, Y. Two-Dimensional MXene-Polymer Heterostructure with Ordered In-Plane Mesochannels for High-Performance Capacitive Deionization. *Angewandte Chemie International Edition* **2021**, *60*, 26528–26534.
30. Cai, Y.; Wang, Y.; Zhang, L.; Fang, R.; Wang, J. 3D Heterostructure Constructed by Few-Layered MXenes with a MoS<sub>2</sub> Layer as the Shielding Shell for Excellent Hybrid Capacitive Deionization and Enhanced Structural Stability. *ACS Appl Mater Interfaces* **2022**, *14*, 2833–2847.
31. Liang, J.; Yu, J.; Xing, W.; Tang, W.; Tang, N.; Guo, J. 3D interconnected network architectures assembled from W18O49 and Ti3C2 MXene with excellent electrochemical properties and CDI performance. *Chemical Engineering Journal* **2022**, *435*, 134922.
32. Bo, Z.; Huang, Z.; Xu, C.; Chen, Y.; Wu, E.; Yan, J.; Cen, K.; Yang, H.; Ostrikov, K. Anion-kinetics-selective graphene anode and cation-energy-selective MXene cathode for high-performance capacitive deionization. *Energy Storage Materials* **2022**, *50*, 395–406.
